# The impact of air pollution on hospitalization for COPD patients in China

**DOI:** 10.1093/eurpub/ckad199

**Published:** 2023-11-15

**Authors:** Chen Chen, Yi Wang, Jinglin Song, Juanjuan Yan

**Affiliations:** School of Public Finance & Economics, Shanxi University of Finance and Economics, Taiyuan, China; School of Public Finance & Economics, Shanxi University of Finance and Economics, Taiyuan, China; Post-Doctoral Research Center, Bank of Chongqing, Chongqing, China; School of Health Services and Management, Shanxi University of Chinese Medicine, Jinzhong, China

## Abstract

**Background:**

With the rapid development of the global economy and the acceleration of urbanization, air pollution has become a major environmental problem threatening human health. There is limited evidence on the acute effects of air pollution on chronic obstructive pulmonary disease (COPD).

**Methods:**

From 2014 to 2019, we collected data on daily admissions for COPD patients from a city in China. We used the generalized additive model together with distributed lag models to fit the associations of air pollutants with hospital admissions.

**Results:**

We observed significant increments in the number of daily admissions (0.086–0.109%) for COPD for a unit range increase in air quality index, ${\mathrm{PM}}_{2.5}$ and ${\mathrm{PM}}_{10}$ over four lag days. The impact of air pollution on the number of daily admissions was mainly reflected in the COPD patients who were hospitalized through outpatient departments and tertiary hospitals.

**Discussion:**

Short-term exposure to outdoor air pollution may induce the occurrence or exacerbation of COPD patients; therefore, government departments should strengthen the management of air pollution, improve supervision and control mechanisms, pay attention to the quality of medical services, and reduce the adverse effects of air pollution on patients' health.

## Introduction

With the rapid development of the global economy and urbanization, environmental pollution problems have arisen, including air pollution, water pollution, noise pollution and soil pollution, among which air pollution has become an important environmental problem that endangers people's health [1, 2]. Globally, long-term exposure to air pollution causes 4.2–8.9 million deaths [3, 4], and this number is increasing. In 2016, approximately 91% of the world’s population lived in an environment that did not meet the air quality guidelines proposed by the World Health Organization. Air pollution in urban, suburban and rural areas led to approximately 4.2 million premature deaths, and approximately 90% of these premature deaths occurred in low-income and middle-income countries. Air pollution has increased significantly in low-and middle-income countries, but air quality monitoring is usually sparse [5]. Compared with developed countries, the economic situation of developing countries generally lags behind, and the corresponding living environment is also worse. As the largest developing country in the world, China has experienced rapid economic growth in the past 30 years, but at the same time, the problem of air pollution has become increasingly serious. According to the 2019 World Health Organization Air Quality Report, as of 2019, approximately 98% of China’s cities have air pollution concentrations higher than the World Health Organization’s standard for air pollution, and 47 cities are still listed as the 100 most polluted cities in the world. Thus, China's air pollution problem is still serious.

Chronic obstructive pulmonary disease (COPD) has always been a major global health problem. It is a lung disease characterized by airflow obstruction. Its occurrence is related to the abnormal inflammatory reaction of the lung to harmful gases or particles [6, 7]. According to World Health Statistics, COPD was estimated to be the 12th cause of disability and the 6th cause of death in 1990. By 2020, it had become the fifth leading cause of disability and the third leading cause of death in the world [8]. Many studies have shown that air pollution increases the health risk of COPD [9–15]. Higher exposure to community air pollutants is associated with worsening respiratory symptoms [16]. For the measurement of health risk, most studies analyzed the changes in hospitalization rate and mortality from a macro perspective and found that the daily hospitalization rate and mortality rate are closely related to air pollution [17–20], but there is a lack of heterogeneity analysis of disease types, medical institution characteristics and patient characteristics. In terms of the selection of air pollution indicators, the literature mostly includes studies of respirable particulate matter, sulfur dioxide and nitrogen dioxide, with less research on fine particulate matter, which may have a greater health impact, and the use of the air quality index (AQI) as a comprehensive indicator, which especially warrants discussion. In addition, most studies on the health effects of air pollution have focused on developed countries, and fewer studies have been conducted in developing countries; however, air quality problems tend to be more severe in developing countries, so people in these countries also experience more health problems due to air pollution [21–24].

This paper used air quality data, inpatient data and medical institution data from a city in China, covering the period from 1 January 2014 to 30 June 2019 and selected COPD as the study object to explore the impact of air pollution on hospitalizations. The contributions of this paper are mainly in the following two aspects: first, the studies on air pollution and the number of admissions are mainly focused on developed countries, and there are fewer microscopic data for developing countries, where air quality problems are more serious. This paper uses a data sample from a city in China with a long-time span and many admissions, to enrich the research in developing countries. Second, this article further analyzes the impact of air pollution on hospitalization rates for different tiers of hospitals.

## Methods

### Study area

The study city of this paper is in southwest China and the upper reaches of the Yangtze River. The total area of the city is 82 400 square kilometers, with 26 districts, 8 counties, 4 autonomous counties and a subtropical monsoonal humid climate. By the end of 2022, the sample city had a GDP of more than 200 billion RMB, ranking among the top 10 of all cities in China. The total resident population in the city was 32.1334 million, with males accounting for 50.5% and females accounting for 49.5% of the population. The population distribution by age group is as follows: 0–14 years old, 14.6%; 15–64 years old, 67.1%; and 65 years and older, 18.3%. Furthermore, the main sources of air pollution in the sample city included emissions from enterprises such as steel, thermal power, cement, and chemical industries, as well as emissions from motor vehicles and ships. By comparing the national macro database and the current commonly used micro database, we found that the data of the sample city are not significantly different from the statistical values of other national databases, and we believe that this sample city may be a representative case for the study of the relationship between air quality and health outcomes.

### Data collection

This paper collected 24-h daily air pollutant monitoring data at the city level from 1 January 2014 to 30 June 2019 from the Ecological Environment Bureau of City A, including AQI, ${\mathrm{PM}}_{2.5}$and ${\mathrm{PM}}_{10}$. First, the AQI (https://www.mee.gov.cn/ywgz/fgbz/bz/bzwb/jcffbz/201203/W020120410332725219541.pdf) is a dimensionless index that quantitatively describes the condition of air quality, and higher values indicate more serious air pollution. AQI is calculated based on daily data for six pollutants, including the 24-h average concentrations of sulfur dioxide (SO_2_), nitrogen dioxide (NO_2_), particulate matter (PM_10_), particulate matter (PM_2_._5_), carbon monoxide (CO), as well as the daily maximum 1-h average and daily maximum 8-h rolling average of ozone (O_3_). The reference standards for AQI classification calculation are the ‘Ambient Air Quality Standards’ (GB3095-2012) and ‘Ambient Air Quality Index Technical Regulations (Trial)’ (HJ633-2012) promulgated by the Ministry of Environmental Protection of China. The reported AQI corresponds to the pollutant with the highest numerical value currently displayed. The index ranges from 0 to 500 and is the most common indicator used to report daily air quality to the public. (The calculation processes of AQI are shown in the supplementary materials.) Second, ${\mathrm{PM}}_{2.5}$ is the particulate matter in ambient air with an aerodynamic diameter of 2.5 microns or less, which is also called fine particulate matter. Its diameter is <1/20 of the thickness of a human hair, and it can remain suspended in the air for a long time. Third, ${\mathrm{PM}}_{10\;}$is particulate matter with a particle size <10 microns. It is the general term for solid and liquid particles floating in the air. It is also called inhalable particulate matter. To adjust the potential mixed effects of weather conditions, we collected meteorological data from city A from 1 January 2014 to 30 June 2019 from the National Meteorological Science Data Center, including daily 24-h mean temperature, relative humidity, atmospheric pressure, precipitation, average wind speed and sunshine duration.

In addition, to measure the impact of air pollution on the number of admissions, the data used in this study were from 10% random samples on inpatient medical record cover sheets in City A of China from 1 January 2014 to 30 June 2019. Referring to the literature, this study selected only patients whose main diseases were diagnosed as COPD (ICD-10: J40-J44 and J47) and removed 94 samples with missing values, yielding a final sample size of 45 967. All the data used in this article were taken from the China Regional Health Information Platform, which collects administrative types of medical data. Importantly, personal information was inaccessible, only objective variables could be analyzed under the restrictive governance of the policy, and data were analyzed at the average level. All research procedures in this paper were approved by the Regional National Health Committee of China.

### Statistical analyses

The generalized additive model (GAM) can analyze the complex nonlinear relationship between the explained and explanatory variables. The model has been widely applied in environmental epidemiology studies to explore the relationship between air pollutant exposure and adverse outcomes. For the total number of people in a region, the occurrence of hospitalization for COPD is a small probability event, and its distribution approximately follows the Poisson distribution. Therefore, we established a GAM based on quasi-Poisson distribution to analyze the impact of the AQI, which measures air quality conditions, and two major air pollutants (${\mathrm{PM}}_{2.5}$ and ${\mathrm{PM}}_{10}$), on the number of admissions for COPD. The main process included the following steps: (i) The cubic spline smoothing function was included in the GAM model to control the long-term trend and time change; (ii) weekends and public holidays were included in the model as dummy variables; and (iii) the natural smoothing functions of meteorological factors such as the mean temperature, relative humidity, atmospheric pressure, precipitation, average wind speed and sunshine duration were added to the model to control the mixed effects of meteorological factors on the direct correlation between air pollution and admissions of COPD. Given that smoothing functions are effective in capturing nonlinear relationships, modeling the different influences of covariates across value ranges, improving model flexibility and predictive capability, and mitigating the impact of outliers and noise, it is appropriate to employ smoothing functions to adjust for the time trend and meteorological factors in model (1). Model (1) is as follows:

(1) $$\begin{array}{c} l\mathrm{og}\left(E_{t}\right)=\alpha +\beta (X_{t})+\mathrm{ns}(\mathrm{Time},\;\mathrm{df})+\mathrm{ns}(T,\;\mathrm{df})\\ +\;\mathrm{ns}(H,\;\mathrm{df})+\mathrm{ns}(P,\;\mathrm{df})+\;\mathrm{ns}(R,\;\mathrm{df})\\ +\;\mathrm{ns}\left(W,\;\mathrm{df}\right)+\mathrm{ns}\left(S,\;\mathrm{df}\right)+\mathrm{Dow}+\mathrm{Holiday}, \end{array}$$

where α is the intercept; *E_t_* was the expected value of the number of admissions for COPD on day t; $X_{t}$ is the explanatory variable for air pollution, including AQI, ${\mathrm{PM}}_{2.5}$ and ${\mathrm{PM}}_{10}$; Time is the date variable; *T* is the mean temperature, *H* is the relative humidity; *P* is the barometric pressure; *R* is the precipitation; *W* is the average wind speed; and *S* is the length of daylight; Dow is the dummy variable for weekend (0 = working day, 1 = weekend); Holiday is the dummy variable for public; ns is a spline smoothing function for the nonlinear variables; and df is the degree of freedom. Based on existing studies, we chose degrees of freedom of 4/year for time and 3 for meteorology variables (*T*, *H*, *P*, *R*, *W*, *S*). We applied polynomial distributed lag models (DLMs) to estimate the cumulative effects of air pollutants along a lag of 3 days (i.e. the present day and the previous 3 days) [23, 25]. Furthermore, we analyzed the association between air pollutants and COPD hospitalizations in subgroups by admission route and tier of hospitals. Moreover, sensitivity analysis was conducted by varying lag days for air pollutants, lag days for meteorological factors, degrees of freedom for meteorological factors to examine the robustness of the results in our study. In addition, we conducted heterogeneity analysis by age stratification, and the relevant results are shown in Supplementary figure S7. All statistical tests were bilateral tests, and a *P* value of <0.05 indicated statistical significance. All methods were performed in accordance with the relevant guidelines and regulations.

## Results

### Descriptive statistics

Table 1 summarizes the descriptive statistics of daily air pollutant concentrations and meteorological conditions in this study. From 1 January 2014 to 30 June 2019, the daily 24-h average AQI in City A was 75, with a 75th percentile value of 88 and a maximum value of 262. According to the Technical Regulations on Ambient AQI (Trial) (HJ633-2012) issued by the Ministry of Environmental Protection of the People's Republic of China, the AQI is divided into six categories: excellent (1–50), good (51–100), mild pollution (101–150), moderate pollution (151–200), heavy pollution (201–300) and severe pollution (>300). Most of the time, the air quality in City A was categorized as good, but there were also cases of heavy pollution. Furthermore, the daily average concentrations of ${\mathrm{PM}}_{2.5}$and ${\mathrm{PM}}_{10}$in City A were 51 (μg/m^3^) and 77 (μg/m^3^), respectively, which were lower than the 24-h average secondary standard concentration limits for pollutants (${\mathrm{PM}}_{2.5}$: 75 μg/m^3^; ${\mathrm{PM}}_{10}$: 150 μg/m^3^) of the Ambient Air Quality Standards (GB3095-2012) issued by the Ministry of Environmental Protection of the People’s Republic of China. However, the maximum daily average concentrations of ${\mathrm{PM}}_{2.5}$and ${\mathrm{PM}}_{10}$ were 211 and 292 μg/m^3^, respectively, indicating that City A experienced severe pollution during the study period. During the study period, the mean temperature was approximately 17°C, the minimum temperature and the maximum temperature were 1°C and 37°C, respectively; the average relative humidity was 76%; the minimum relative humidity and the maximum relative humidity were 37% and 97%, respectively; the average air pressure was 985 hPa, the lowest and highest air pressure were 963 and 1012 hPa; the average precipitation was 3 mm, and the maximum precipitation was 111 mm; the average wind speed was 1 m/s, the minimum wind speed and the maximum wind speed were 0 and 4 m/s, respectively; and the average sunshine duration was approximately 3 h, and the maximum sunshine duration was 13 h.

**Table 1:** Descriptive statistics of daily air pollutant concentrations and weather conditions in this study

	Mean	SD	Min	P25	P50	P75	Max
AQI	75	39	14	49	65	88	262
Pollutant (μg/m^3^)							
${\mathrm{PM}}_{2.5}$	51	32	7	28	41	63	211
${\mathrm{PM}}_{10}$	77	43	12	46	68	97	292
Weather conditions							
Temperature (°C)	17	8	1	11	17	23	37
Humidity (%)	76	11	37	69	78	85	97
Pressure (hPa)	985	8	963	978	985	991	1012
Precipitation (mm)	3	9	0	0	0	2.1	111
Wind (m/s)	13	4	3	11	13	15	37
Sunshine (hours)	2	4	0	0	0	5	13

**Figure 1:**
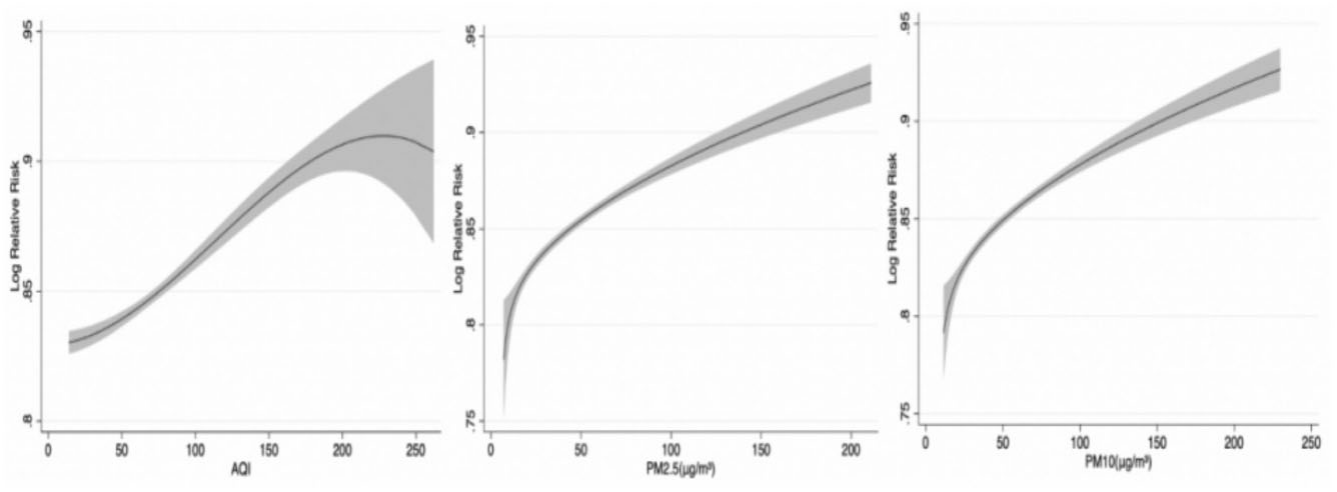
Does-response relationships between air pollutants and COPD hospitalization. (The *X*-axis represented the concentration of air pollutants at the lag of three days, the *Y*-axis indicated Log relative risk of COPD hospitalization. The shaded areas were the 95% confidence interval.)

Figure 1 illustrates the dose–response associations between air pollutants (AQI, ${\mathrm{PM}}_{2.5}$,${\;\mathrm{PM}}_{10}$) with a lag of 3 days (lag0–lag3) and the relative risk of COPD hospitalization. The curves associated with AQI, ${\mathrm{PM}}_{2.5}$ and ${\mathrm{PM}}_{10}$ presented similar trends, which indicated that a higher concentration of air pollutants might cause a significant increase in the relative risk of COPD hospitalization.

### Regression results

**Figure 2:**
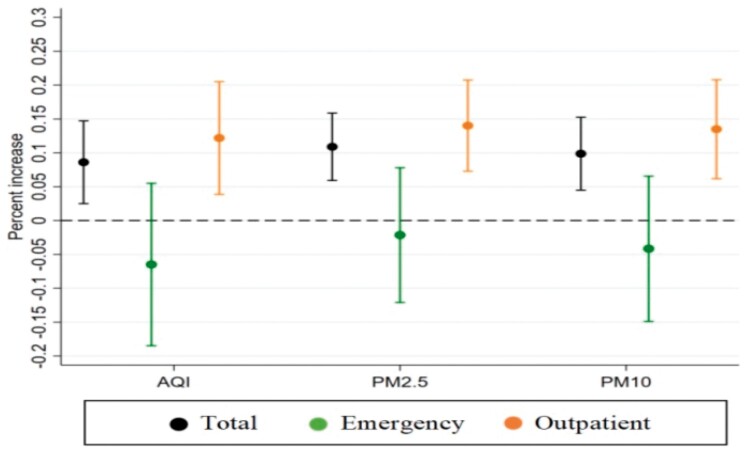
Cumulative percentage increase in daily admissions for COPD associated with a unit increase in air pollutant concentrations over a lag of 0–3 days

Figure 2 summarizes the estimated associations between air pollutants and COPD admissions. After adjusting for the confounding effects of meteorological factors, holidays and weekends, the impacts of AQI, ${\mathrm{PM}}_{2.5}$ and ${\mathrm{PM}}_{10}$ on COPD admissions had a significant positive correlation (*P* < 0.05). For a unit increase in AQI, the cumulative increase in daily admissions due to COPD over lags of 0–3 days was 0.086%. For a 1 μg/m^3^ increase in ${\mathrm{PM}}_{2.5}$ and ${\mathrm{PM}}_{10}$, the cumulative increases in daily admissions due to COPD over lags of 0–3 days were 0.109% and 0.1%, respectively. From the results of admission route, there were nonsignificant associations of all pollutants with emergency department admissions due to COPD. However, figure 2 shows the associations between air pollutants and outpatient department admissions due to COPD, which had similar patterns of association as the full sample. For a unit increase in AQI, the cumulative increase in daily outpatient department admissions due to COPD over lags of 0–3 days was 0.122%. For a 1 μg/m^3^ increase in ${\mathrm{PM}}_{2.5}$ and ${\mathrm{PM}}_{10}$, the cumulative increases in daily outpatient department admissions due to COPD over lags of 0–3 days were 0.14% and 0.135%, respectively.

**Figure 3:**
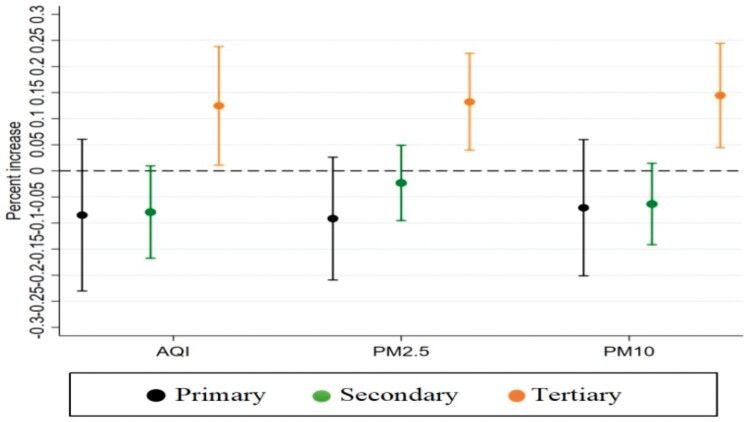
Cumulative percentage increase in daily admissions for COPD at different tiers of hospitals associated with a unit increase in air pollutant concentrations over a lag of 0–3 days

Figure 3 summarizes the estimated associations between air pollutants and COPD admissions in different tiers of hospitals. After adjusting for the confounding effects of meteorological factors, holidays and weekends, there were nonsignificant associations of all pollutants with admissions to primary and secondary hospitals due to COPD. In contrast, there was a significant positive correlation (*P* < 0.05) between AQI, ${\mathrm{PM}}_{2.5}$,$\;{\mathrm{PM}}_{10}$ and admissions to tertiary hospitals due to COPD. For a unit increase in AQI, the cumulative increase in daily admissions to tertiary hospitals due to COPD over lags of 0–3 days was 0.125%. For a 1 μg/m^3^ increase in ${\mathrm{PM}}_{2.5}$ and ${\mathrm{PM}}_{10}$, the cumulative increases in daily admissions to tertiary hospitals due to COPD over lags of 0–3 days were 0.132% and 0.145%, respectively. From the results of admissions to different tiers of hospitals, we found that the impact of air pollution on the number of admissions due to COPD mainly occurred in tertiary hospitals, while primary and secondary hospitals were not significantly affected.

### Sensitivity analysis

To further verify the stability of the results, we conducted additional analyses. First, we varied the lag days for air pollutants (AQI, ${\mathrm{PM}}_{2.5}$, ${\mathrm{PM}}_{10}$) from lag0 to lag6 to assess the stability of the results. Second, we altered the lag days for meteorological factors (i.e. daily 24-h mean temperature, relative humidity, atmospheric pressure, precipitation, average wind speed and sunshine duration) from lag0 to lag3 to examine the sensitivity of the results to changes in meteorological factors. Third, we varied the degrees of freedom for time (7/12 per year) and meteorological factors (4–6) to examine the robustness of the results. The results (Supplementary figures S1–S6 and Table S1) support the stability of the findings.

## Discussion

In this paper, we used air quality data, inpatient data and medical institution data from a city in China, covering the period from 1 January 2014 to 30 June 2019, and selected COPD as the study object. We used a GAM together with DLMs and linear models to explore the impact of air pollution on hospitalizations and medical expenditures. After adjusting for the confounding effects of meteorological factors, holidays and weekends, the impacts of AQI, ${\mathrm{PM}}_{2.5}$ and ${\mathrm{PM}}_{10}$ on COPD admissions had a significant positive correlation (*P* < 0.05). Patients with COPD who were hospitalized through outpatient departments and tertiary hospitals were more likely to be affected by air pollutants. Our findings suggest that healthcare providers should be more proactive in preventing and treating COPD, encouraging patients to take protective measures, developing personalized care plans, and recommending patients to primary care facilities to reduce the pressure on tertiary care facilities.

In epidemiological studies, mortality is usually selected to measure the impact of air pollution on health. Compared with mortality, morbidity can be a more sensitive response to exposure to air pollutants. Our findings showed that for each unit increase in AQI, the cumulative increase in daily admissions due to COPD over lags of 0–3 days was 0.086%. For a 1 μg/m^3^ increase in ${\mathrm{PM}}_{2.5}$ and ${\mathrm{PM}}_{10}$, the cumulative increases in daily admissions due to COPD over lags of 0–3 days were 0.109% and 0.1%, respectively. These results were consistent with the findings of most studies [26]. Luo et al. [27] studied the short-term effects of air pollution on respiratory disease hospitalization in Taiyuan, China, and found that a 10-µg/m^3^ increment in ${\mathrm{PM}}_{2.5}$ at lag0 was mostly strongly associated with a 0.547% increment in COPD hospitalization, and the effects in multiday lags were greater. Liu et al. [28] studied the impact of air pollution in Jinan on hospitalization behavior and found that ${\mathrm{PM}}_{2.5}$ in urban areas increased by 10 µg/m^3^, urban emergency departments for respiratory diseases increased by 1.4%, and suburban emergency departments increased by 1.5%. Li et al [29] analyzed the effect of ${\mathrm{PM}}_{2.5}$ on hospitalization rates for COPD and found that a 10 µg/m^3^ increase in ${\mathrm{PM}}_{2.5}$ lagged by 0–7 days may lead to increases of 3.1% in the number of admissions due to COPD.

Our findings indicated that the impact of air pollution on the number of COPD admissions varies across different hospitalization types. Specifically, there was a significant positive correlation between outpatient admissions for COPD patients and air pollution, whereas there was no significant association between emergency admissions for COPD patients and air pollution. In addition, we found differences in the admission rates of COPD patients among healthcare institutions of different tiers, with two main reasons: first, from the perspective of healthcare service supply, the distribution of medical resources at all tiers of hospitals in China is relatively unbalanced, and tertiary hospitals are the highest level hospitals in China, with more high-quality medical services and technical level, and have advantages in scientific research and equipment; second, from the perspective of healthcare service demand, Chinese patients have the freedom to choose healthcare institutions of different tiers for treatment. The healthcare insurance reimbursement system also supports patients in freely choosing healthcare facilities. Therefore, we found that air pollution had the greatest impact on admissions of COPD in tertiary hospitals, which is consistent with the behavior of Chinese people who had a preference to be admitted to high-level hospitals [24].

Our study had several limitations. First, as in most previous time-series studies, we simply obtained data on air pollution and meteorological factors in cities, which may differ from individual exposure levels and cannot represent the total exposure of the population [30–32]. Therefore, our study may underestimate the impact of AQI, ${\mathrm{PM}}_{2.5}$ and ${\mathrm{PM}}_{10}$ on hospitalization for COPD [33]. Second, the data we collected included only one disease type, chronic pulmonary resistance, which reduced the universality of our results for respiratory diseases. Third, personal exposure to air pollution includes both environmental and indoor pollution, and our study lacked an analysis of indoor pollution. Finally, the occurrence of COPD is also influenced by factors such as smoking history and occupational exposure, which were not considered.

## Supplementary Material

ckad199_Supplementary_Data

## Data Availability

This article is based on data provided with permission from the China regional health information center and shared data with the corresponding author. Key points Generalized additive model together with distributed lag models to fit the associations of air pollutants with hospital admissions for COPD patients. On average, positive correlation between air pollutants level and COPD admissions had a significant positive correlation. COPD patients attending outpatient departments and tertiary hospitals are more affected by air pollution.
