# Supplementary material for: The impact of air pollution on hospitalization for COPD patients in China
Source: Eur J Public Health. 2023 Nov 15;34(1):150–5. doi: 10.1093/eurpub/ckad199 (PMC10843963; doi:10.1093/eurpub/ckad199)
Supplement: ckad199_Supplementary_Data [file ckad199_supplementary_data.pdf]

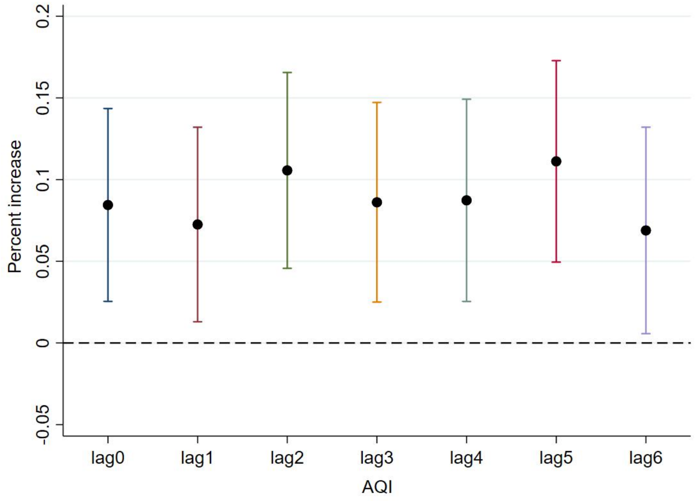

Figure S1. Cumulative percentage increase in daily admissions for COPD associated with a unit increase in AQI over different lag days.

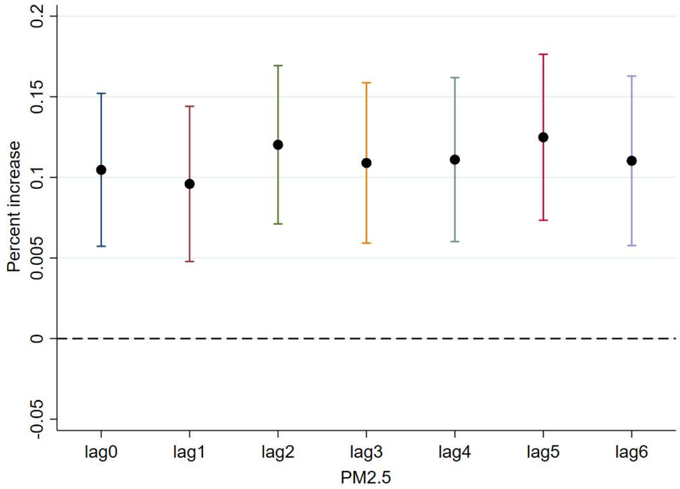

Figure S2. Cumulative percentage increase in daily admissions for COPD associated with a unit increase in $PM_{2.5}$ over different lag days.

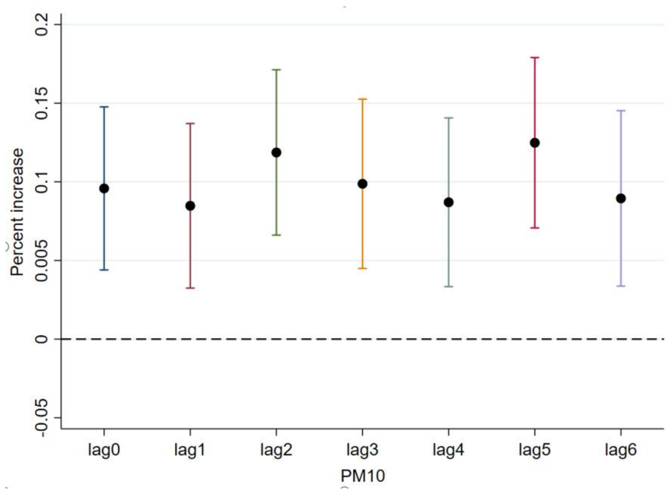

Figure S3. Cumulative percentage increase in daily admissions for COPD associated with a unit increase in PM_{10} over different lag days.

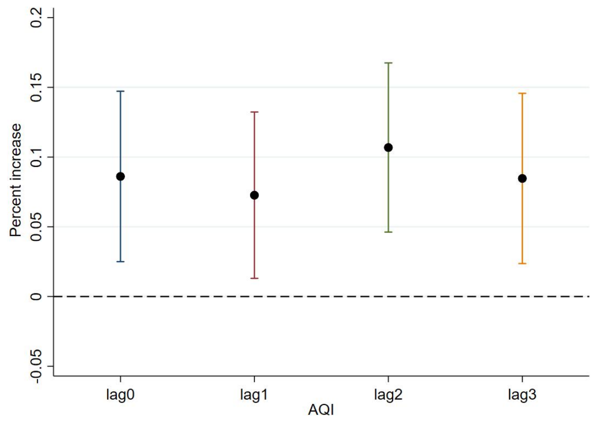

Figure S4. Cumulative percentage increase in daily admissions for COPD associated with a unit increase in AQI over different lag days of meteorological factors.

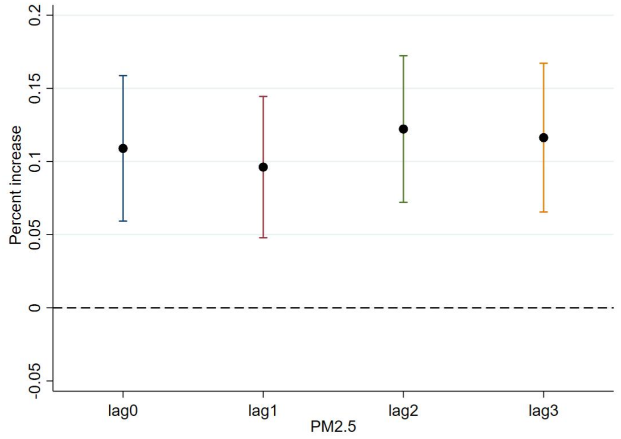

Figure S5. Cumulative percentage increase in daily admissions for COPD associated with a unit increase in $PM_{2.5}$ over different lag days of meteorological factors.

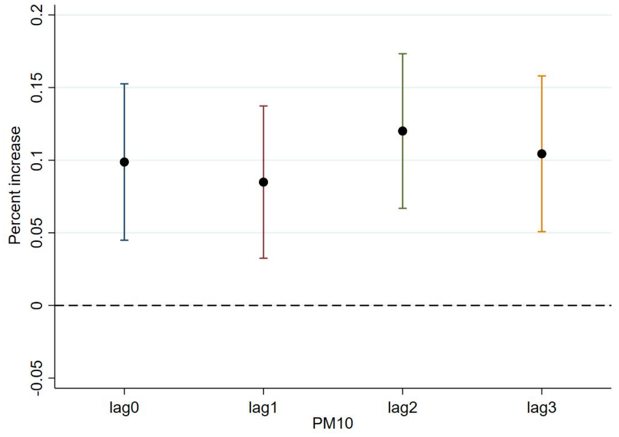

Figure S6. Cumulative percentage increase in daily admissions for COPD associated with a unit increase in PM_{10} over different lag days of meteorological factors.

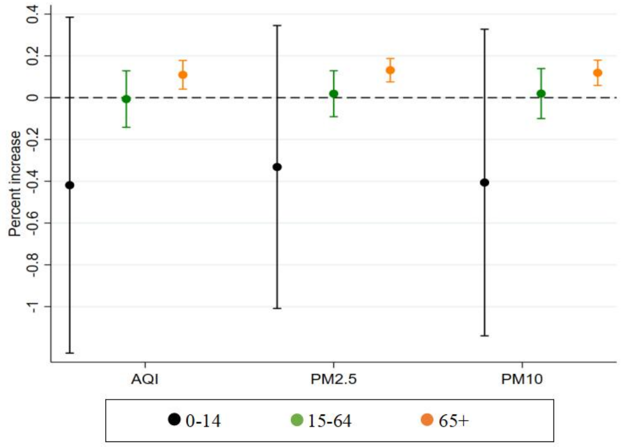

Figure S7. Cumulative percentage increase in daily admissions for COPD at different age stratifications associated with a unit increase in air pollutant concentrations over a lag of zero to three days.

Table S1. Sensitivity analysis of air pollution on hospitalizations of COPD patients by changing df=7 and 12 per year of time, df=4-6 of meteorological factors in China (health effects were showed as percent changes and 95% CI with per a unit increment in air pollutant concentrations).

Variables	AQI	$PM_{2.5}$	PM_{10}
Time			
df=7/year	0.074**	0.104***	0.085***
	(0.040,0.108)	(0.079,0.129)	(0.058,0.112)
df=12/year	0.070**	0.099***	0.082***
	(0.038,0.102)	(0.075,0.123)	(0.056,0.108)
Meteorological factors			
df=4	0.086***	0.108***	0.088***
	(0.055,0.117)	(0.082,0.134)	(0.060,0.116)
df=5	0.086***	0.102***	0.084***
	(0.055,0.117)	(0.078,0.126)	(0.057,0.111)
df=6	0.082***	0.102***	0.083***
	(0.052,0.112)	(0.078,0.126)	(0.056,0.110)

* $p < 0.1$, ** $p < 0.05$, *** $p < 0.01$

The calculation processes of AQI :

Step 1:

The individual air quality index for pollutant P is calculated according to equation (1):

$$IAQI_P = \frac{IAQI_{Hi} - IAQI_{Lo}}{BP_{Hi} - BP_{Lo}}(C_P - BP_{Lo}) + IAQI_{Lo} \qquad (1)$$

In equation (1):

$IAQI_P$ —Individual air quality index of pollutant P.

C_P —The mass concentration value of Pollutant P.

BP_{Hi}—The upper limit of the concentration threshold for pollutants in table S2 that is close to C_P.

BP_{Lo}—The lower limit of the concentration threshold for pollutants in table S2 that is close to C_P.

$IAQI_{Hi}$—The individual air quality index corresponding to BP_{Hi} in table S2.

$IAQI_{Lo}$—The individual air quality index corresponding to BP_{Lo} in table S2.

Table S2 Individual air quality index and pollutant concentration limits

IAQI	Pollutant Concentration Limits									
	**SO_2 24-hour average (μg/m3)**	**SO_2 1-hour average (μg/m3)**	**NO_2 24-hour average (μg/m3)**	**NO_2 1-hour average (μg/m3)**	**PM_{10} 24-hour average (μg/m3)**	**CO 24-hour average (mg/m3)**	**CO 1-hour average (mg/m3)**	**O_3 1-hour average (μg/m3)**	**O_3 8-hour rolling average (μg/m3)**	**$PM_{2.5}$ 24-hour average (μg/m3)**
0	0	0	0	0	0	0	0	0	0	0
50	50	150	40	100	50	2	5	160	100	35
100	150	500	80	200	150	4	10	200	160	75
150	475	650	180	700	250	14	35	300	215	115
200	800	800	280	1200	350	24	60	400	265	150
300	1600	(2)	565	2340	420	36	90	800	800	250
400	2100	(2)	750	3090	500	48	120	1000	(3)	350
500	2620	(2)	940	3840	600	60	150	1200	(3)	500
Note:	(1)The 1-hour average concentration limits of SO_2、NO_2 and CO are only used for real-time reporting. The 24-hour average concentration limits of the corresponding pollutants need to be used in daily reports.<br>(2) If the 1-hour average concentration value of SO_2 is higher than 800 μg/m3, the air quality sub-index will no longer be									

	calculated. The SO_2 air quality sub-index will be reported based on the sub-index calculated based on the 24-hour average concentration.<br>(3) If the 8-hour average concentration of O_3 is higher than 800 μg/m3, the air quality sub-index will no longer be calculated. The O_3 air quality sub-index will be reported based on the sub-index calculated from the 1-hour average concentration.<br>(4) IAQI: Individual air quality index.

Step 2:

The air quality index is calculated according to equation (2):

$$AQI = \max\left\{IAQI_1, IAQI_2, IAQI_3, \cdots, IAQI_n\right\} \quad (2)$$

In equation (2):

IAQI —Individual air quality index.

n —Pollutant.
